# Ultrasound-Assisted Deep Eutectic Solvent Extraction of Antioxidant and Anti-Colorectal Cancer Proteins from *Spirulina* Biomass: Process Intensification, Characterization, and Bioactivity Evaluation

**DOI:** 10.3390/antiox14030365

**Published:** 2025-03-19

**Authors:** May Thu Zin, Thida Kaewkod, Supakit Chaipoot, Gochakorn Kanthakat, Yan-Yu Chen, Benjamas Cheirsilp, Sirasit Srinuanpan

**Affiliations:** 1 Master of Science Program in Applied Microbiology (International Program), Department of Biology, Faculty of Science, Chiang Mai University, Chiang Mai 50200, Thailand; maythu_zin@cmu.ac.th; 2 Department of Biology, Faculty of Science, Chiang Mai University, Chiang Mai 50200, Thailand; thida.kaewkod@cmu.ac.th; 3 Microbial Biorefinery and Biochemical Process Engineering Research Group, Chiang Mai University, Chiang Mai 50200, Thailand; 4 Department of Chemical Engineering, National Chung Hsing University, Taichung 40227, Taiwan; annayyc0707@nchu.edu.tw; 5 Center of Excellence in Microbial Diversity and Sustainable Utilization, Chiang Mai University, Chiang Mai 50200, Thailand; supakit.ch@cmu.ac.th; 6 Multidisciplinary Research Institute, Chiang Mai University, Chiang Mai 50200, Thailand; 7 Faculty of Agro-Industry, Chiang Mai University, Chiang Mai 50100, Thailand; gochakorn.g@gmail.com; 8 Center of Excellence in Innovative Biotechnology for Sustainable Utilization of Bioresources, Faculty of Agro-Industry, Prince of Songkla University, Hat Yai 90110, Thailand; benjamas.che@cmu.ac.th; 9 Office of Research Administration, Office of the University, Chiang Mai University, Chiang Mai 50200, Thailand

**Keywords:** antioxidants, anticancer, deep eutectic solvents, extraction, protein, *Spirulina*, ultrasound

## Abstract

*Spirulina*, a cyanobacterial biomass, is renowned for its high protein content and bioactive compounds, making it a promising candidate for health-promoting applications. This study explores the ultrasound-assisted deep eutectic solvent (DES) extraction technique for isolating antioxidants and anticancer proteins from *Spirulina* biomass, aiming to enhance extraction efficiency and preserve protein bioactivity. The extraction process was optimized using response surface methodology (RSM), varying parameters such as biomass concentration, sonication amplitude, and extraction duration. The optimized extraction conditions—5% biomass concentration, 40% sonication amplitude, and 22-minute extraction—achieved a high protein yield of 80.62%, with a protein concentration of 442.88 mg/g extract and an essential amino acid content of 39.91%. The extracted proteins exhibited remarkable bioactivity, including strong antioxidant properties, with 2,2-diphenyl-1-picrylhydrazyl (DPPH) radical scavenging activity of 0.25 mg GAE/g, 2,2′-azino-bis(3-ethylbenzthiazoline-6-sulphonic acid) (ABTS) radical scavenging activity of 0.58 mg TE/g, and ferric reducing antioxidant power (PFRAP) of 9.63 mg gallic acid equivalent (GAE)/g. Additionally, the protein extract displayed selective cytotoxicity against colorectal cancer cell lines, with half-maximal inhibitory concentration (IC_50_) values of 10.25 mg/mL for Caco-2 and 15.40 mg/mL for HT-29 cells, while maintaining low toxicity towards normal Vero cells. Apoptosis rates of 70.43% in Caco-2 and 51.33% in HT-29 cells further confirm the anticancer potential of the extract. The functional properties of the extracted protein, including high foaming capacity (100%), emulsifying capacity (94.05%), and digestibility (85.77%), underscore its potential for diverse applications in food, pharmaceutical, and nutraceutical industries. This eco-friendly and efficient extraction approach aligns with sustainable development goals and offers a viable strategy for harnessing *Spirulina*’s bioactive potential.

## 1. Introduction

Food production faces growing challenges, with rising demand necessitating sustainable protein sources. While animal-based proteins have long dominated the field, concerns over sustainability and health impacts have fueled interest in plant-based alternatives. Traditionally, soy, legumes, and oilseeds have been key sources, but algae proteins are emerging for their superior nutritional, functional, and bioactive properties [1]. Algae, including microalgae and cyanobacteria (blue-green algae), offer rapid growth, high photosynthetic efficiency, low water use, and do not require arable land, making them a promising sustainable option. They not only provide high protein content but also feature an impressive amino acid profile, rich in essential amino acids recommended by the World Health Organization (WHO). Several algae and cyanobacteria species, including *Arthrospira* (*Spirulina*), *Chlorella*, *Dunaliella*, and *Haematococcus*, have been approved for human consumption by the European Food Safety Authority [2]. Among these, *Spirulina*, a cyanobacterium, stands out for its exceptionally high protein content, which constitutes 50–70% of its dry weight [3]. Additionally, *Spirulina* is abundant in bioactive compounds such as phycocyanins, polysaccharides, and vitamins, which significantly contribute to its nutritional and therapeutic potential. Studies highlight *Spirulina*’s antioxidant, anti-inflammatory, and anticancer properties, making it valuable in combating oxidative stress and related diseases. With colorectal cancer among the most prevalent, natural compound-based therapies are gaining traction. Algal proteins, particularly from *Spirulina*, show promise in inhibiting cancer cell proliferation and inducing apoptosis, with effects observed in lung (A549), ovarian (SKOV3), cervical (HeLa), and breast (MCF7) cancer cell lines [4,5]. Despite these promising findings, significant challenges remain in optimizing extraction techniques and fully harnessing the therapeutic potential of *Spirulina* proteins. The entire algal biomass may contain antinutritional components, such as specific phenolic compounds, which can hinder protein absorption and utilization.

Extracting proteins from Spirulina is challenging due to its robust cell wall and complex protein–lipid matrix, which hinder protein release. Conventional solvent-based methods pose environmental concerns, yield limitations, and protein degradation risks. Techniques like acid precipitation and alkaline extraction, though simple, contribute to equipment corrosion and industrial effluents, making them less suitable for modern sustainable applications [6]. Developing high-quality, cost-effective, and environmentally friendly protein extraction techniques remain an essential focus for both research and industry. In this context, deep eutectic solvents (DESs) have emerged as environmentally friendly alternatives to traditional organic solvents. Recently, DESs have gained attention as promising agents for protein extraction [7]. These solvents are created by combining hydrogen bond donors and acceptors, forming eutectic mixtures that remain liquid at room temperature. DESs offer several advantages, including biodegradability, low toxicity, minimal volatility, negligible vapor pressure, and non-flammability, making them highly preferred in sustainable extraction processes [8]. Choline chloride (ChCl) is the most used base component in DES formulations for protein extraction studies. Various DES combinations, such as choline chloride with glycerol, ethylene glycol, 1,4-butanediol, 1,2-butanediol, and polyethylene glycol, have been reported to enhance protein yields by 10–50% compared to conventional alkaline extraction methods [9]. Challenges associated with traditional protein extraction and modification techniques have spurred the exploration of innovative methods. Among these, ultrasound-assisted extraction (UAE) has been widely studied for its ability to improve the physical and functional properties of extracted proteins. By integrating DES with emerging techniques like UAE, researchers aim to develop efficient, sustainable, and high-yield protein extraction methods.

Ultrasound enhances protein extraction by generating mechanical waves that induce acoustic cavitation, leading to mechanical, thermal, and chemical effects [6]. This process breaks down cell walls and improves mass transfer, increasing protein yields. Cost-effective and widely used in the food industry, ultrasound extraction employs baths and probes, with probes being more efficient due to their focused ultrasonic intensity [10]. Ultrasound treatment not only increases the yield but also accelerates the rate of protein extraction while influencing the functional properties of the proteins. Purdi et al. [11] demonstrated that UAE can significantly enhance protein yields compared to conventional methods. Their study reported a UAE-derived protein yield of 76.83%, in contrast to only 32.48% from conventional extraction without ultrasound. This substantial difference highlights the efficiency of UAE in protein extraction from *Spirulina*. Similarly, Braspaibon et al. [12] found that ultrasound-assisted alkaline extraction (UAAE) of *Spirulina* at 60% amplitude, 30 min sonication, and pH 12 resulted in a protein yield of 81.86%, which is generally higher than yields obtained through conventional extraction methods. These findings emphasize the advantages of UAE in enhancing protein recovery. The effectiveness of UAE is influenced by several extraction parameters, including sonication power, duration, temperature, pH, and the solid-to-solvent ratio, as well as the specific protein source [9,10,11,12]. Careful optimization of these conditions is essential to achieve the highest yield and preserve protein functionality.

The choice of solvent plays a crucial role in determining the efficiency of protein extraction. The combination of DES with sonication for protein extraction from algal biomass is rarely reported, yet it holds significant potential to enhance extraction yields while reducing extraction time. Integrating DES with UAE offers a synergistic approach that can overcome the limitations of traditional extraction methods. DES helps maintain protein integrity by minimizing chemical degradation during the extraction process, while UAE shortens extraction times, reducing the risk of thermal or oxidative damage to sensitive proteins. DESs provides an eco-friendly, biodegradable, and customizable solvent system, while UAE enhances extraction efficiency through cavitation effects. Together, these methods address the shortcomings of conventional extraction techniques, including environmental concerns, low yields, and protein degradation. In addition to protein extraction, the combined approach is also effective in recovering bioactive compounds, such as antioxidants and pigments, expanding its potential applications in the health, nutrition, and pharmaceutical sectors. Despite these advancements, research exploring the combined use of DES and UAE for extracting bioactive compounds, including proteins and antioxidants from algae, remains limited.

Therefore, this study aims to optimize DES-UAE conditions for protein extraction from *Spirulina* biomass and assess its potential for therapeutic applications. Specifically, the research investigates the anticancer activity of *Spirulina*-derived proteins using human colorectal cancer cell lines, Caco-2 and HT-29, which are widely recognized as relevant models for studying colorectal cancer. Additionally, the study evaluates the physicochemical characteristics and functional properties of the extracted proteins.

## 2. Materials and Methods

### 2.1. Algal Biomass

Protein was extracted from the dried biomass powder of the cyanobacterium *Limnospira platensis* (referred to as *Spirulina* biomass in this study), formerly classified as a blue-green alga, purchased from Boonsom Farm, Green Diamon Company Limited (Lot C261067WW-000023, Mae Wang, Chiang Mai, Thailand).

### 2.2. Preparation of Extraction Solvent

The deep eutectic solvent was prepared by adapting the method described by Sharma et al. [6]. Choline chloride functioned as the hydrogen bond acceptor (HBA), while urea served as hydrogen bond donors (HBDs). In brief, the mixture was created at a molar ratio of 1:2 (HBA to HBD), with distilled water added to maintain the same molar ratio and heated at 80 °C under the condition of 3000 rpm stirring until a clear solution was obtained. The prepared deep eutectic solvent was stored in a moisture-controlled cabinet until needed for subsequent testing.

### 2.3. RSM Optimization of Protein Extraction via Integrated DES Extraction and Ultrasound-Assisted Process

*Spirulina* biomass was mixed with a defined amount of DES and sonicated to enhance protein extraction. Ultrasonication was performed using an ultrasonic processor (VC505, Sonics & Materials, Inc., Newtown, CT, USA) fitted with a 6 mm diameter probe. The device operated at a frequency of 20 kHz, with a 40% amplitude setting and a power output of 500 W. To prevent a rapid temperature increase, the reaction chamber was placed in an ice bucket, and a power on/off pulse cycle of 30/30 s was employed. The sample temperature immediately after sonication ranged from 25 to 30 °C. Response surface methodology (RSM) was conducted using Design Expert 7.0 tools to optimize the biomass to sample concentration (%), amplitude (%) and extraction time (minutes) for enhancing protein yield (% of dry weight) in *Spirulina* biomass. The extraction parameters were determined based on previous studies on DES-based and ultrasound-assisted protein extraction [9,10,11]. The central composite design (CCD) was employed to design and study the interaction among three independent variables: sample concentration, amplitude and time. Sample concentration ranged from 1 to 5%, while amplitude and time ranged from 20 to 40% and 15 to 45 min, respectively. RSM with CCD was chosen because it allows for efficient and comprehensive exploration of the parameter space, helping to identify optimal conditions with fewer experimental runs. This method also provides a quadratic model to describe the relationship between the variables and the response, which is essential for optimizing the extraction process and ensuring reproducibility [13]. A second-order polynomial quadratic equation was fitted to analyze the main effects and interactions of each independent variable on the response using the following Equation (1).

(1) $$Y=\beta_{0}+\Sigma \beta_{i}x_{i}+\Sigma \beta_{\mathrm{ii}}x_{i}^{2}+\Sigma \beta_{\mathrm{ij}}x_{i}x_{j}$$

In this equation, the experimental response Y represents the protein yield. The factors or variables are indicated as x_i_ and x_j_. The terms β_0_, β_i_, β_ij_, and β_ii_ correspond to the intercept, linear coefficient, interaction coefficient for the first-order terms, and the squared coefficient, respectively. The model’s fit quality was assessed using the coefficient of determination (R^2^) and analysis of variance (ANOVA). To determine the optimal conditions, response surface plots were created by varying two variables while keeping the other two constant.

### 2.4. Characterization of Extracted Proteins

#### 2.4.1. Determination of Protein Content

The protein content in the crude extract was analyzed using the Lowry method [14]. To prepare the standard protein solution, 10 mg of bovine serum albumin (BSA) was dissolved in 1 mL of phosphate buffer. This solution was subsequently diluted into 5 concentrations: 0.62, 1.25, 2.5, 5, and 10 mg/mL. A standard curve was constructed by plotting the absorbance readings against these concentrations. Protein isolates obtained through ultrasound-assisted extraction were prepared at the same concentration as the BSA standard (10 mg/mL), and the absorbance was recorded at 595 nm using a spectrophotometer.

#### 2.4.2. Determination of Molecular Weight by SDS-PAGE Gel Electrophoresis

The molecular weight distribution of the extracted proteins was determined using SDS-PAGE gel electrophoresis. To prepare the resolving gel, 3 mL of 30% bis-acrylamide, 1.5 mL of 1.5 M Tris-HCl buffer (pH 8.8), 60 µL of 10% SDS, 1.42 mL of deionized water, and 20 µL of TEMED were combined. Polymerization was initiated by adding ammonium persulfate, and the gel was allowed to set for 15–30 min. Once the resolving gel had polymerized, the stacking gel was prepared by mixing 320 µL of 30% bis-acrylamide, 0.5 mL of 0.5 M Tris-HCl buffer (pH 6.8), 20 µL of 10% SDS, 1.26 mL of deionized water, and 5 µL of TEMED, with ammonium persulfate added last. The stacking gel was applied on top and left to polymerize overnight. Molecular weight markers, Precision Plus Protein™ Dual Color Standards (Bio-Rad, Hercules, CA, USA), covering a range of 2 kDa to 250 kDa, were used to reference the protein bands. The electrophoresis process was performed at a constant voltage of 120 V for 120 min, following the methodology described by Htoo et al. [15].

#### 2.4.3. Amino Acid Composition

The amino acid composition of the extracted proteins was analyzed with a modified method based on Pekkoh et al. [16]. To prepare the samples, they were mixed with hexane at a ratio of 1:10 (*w*/*v*) and left to stand overnight. Afterward, the hexane was removed through filtration, and the samples were dried. One gram of the extracted protein was then combined with 6 M HCl and purged with nitrogen gas for one minute. The mixture underwent hydrolysis at 97 °C for 24 h to liberate the amino acids. Following hydrolysis, the sample’s pH was adjusted to 7.0, and 0.5 M trichloroacetic acid (TCA) was added at a 2:1 (*v*/*v*) ratio. The resulting mixture was centrifuged at 10,000 rpm for 5 min, and the supernatant was collected. Before analysis, the supernatant was combined with 0.3 M NaOH. Amino acid profiling was conducted using high-performance liquid chromatography (HPLC) with post-column derivatization method of Shimadzu protocol described by Somjai et al. [17]. The analysis utilized a Shim-pack Amino-Na column (100 mm length × 6.0 mm internal diameter, 5 µm particle size, part no. 228-18837-91, Shimadzu, Kyoto, Japan) and a Prominence RF-20A fluorescence detector (Shimadzu, Japan). The mobile phase comprised sodium citrate buffers with pH values of 3.23 (A) and 10.0 (B), along with a 0.2 M NaOH aqueous solution as phase C. Derivatization of amino acids was performed pre-column using N-Acetyl-L-cysteine and OPA as reagents. The analysis was carried out at a column temperature of 60 °C with a flow rate of 0.4 mL/min and a 10 µL injection volume. Amino acid composition was determined by comparing retention times of the samples with those of a standard amino acid mixture (Sigma, St. Louis, MO, USA).

#### 2.4.4. Determination of Impurities

##### Total Phenolic Content

The total phenolic content (TPC) was measured using the Folin–Ciocalteu reagent (LOBA Chemie, Mumbai, India), following the procedure described by Lomakool et al. [18]. Protein samples at various concentrations were analyzed in a 96-well plate, and a standard curve was generated using gallic acid (GA) solutions with concentrations ranging from 0.02 mg/mL to 0.2 mg/mL. For each assay, 20 μL of the protein sample was combined with 100 μL of 10% Folin–Ciocalteu reagent and allowed to react at room temperature for 5 min. Then, 80 μL of 5% sodium carbonate solution was added, and the mixture was incubated for 1 h. The absorbance was recorded at 765 nm, and the TPC was calculated using the following Equation (2).

TPC (mg-GAE/g-extracted protein) = Absorbance at 1 of GA/Absorbance at 1 of extracted protein(2)

##### Total Sugar Content

The sugar content in the extracted protein was analyzed using the phenol-sulfuric acid method, as outlined by Zin et al. [19]. A glucose standard curve was generated with concentrations ranging from 0.01 to 0.25 mg/mL. For the assay, 0.5 mL of each *Spirulina* extract or glucose solution was combined with 0.5 mL of 5% phenol, followed by the addition of 2.5 mL of 96% sulfuric acid. The reaction was allowed to proceed at room temperature for 10 min. A blank sample containing distilled water was used as a control. The sugar concentration was determined by measuring the absorbance at 490 nm.

### 2.5. Antioxidant Properties

#### 2.5.1. 2-Diphenyl-1-picrylhydrazyl (DPPH) Radical Scavenging Assay

The DPPH radical scavenging activity of the sample was evaluated following a modified procedure based on Lomakool et al. [18]. Gallic acid was used as the reference standard. A 20 mg portion of the sample was dissolved in 1 mL of phosphate buffer. In a 96-well microplate, 50 μL of the sample solution was mixed with 150 μL of a methanolic DPPH solution (0.325 mM). The mixture was incubated at room temperature in the dark for 30 min. After incubation, the absorbance was measured at 517 nm using a microplate spectrophotometer. The half-maximal inhibitory concentration (IC_50_) was determined as the sample concentration that reduced DPPH radical activity by 50%. To calculate the IC_50_, the percentage of inhibition was plotted against various sample concentrations, generating an inhibition curve. The IC_50_ value was obtained by locating the concentration corresponding to 50% inhibition on the curve. The DPPH radical scavenging activity was expressed as gallic acid equivalent (GAE) per gram of extract, using the following Equation (3).

DPPH activity (mg-GAE/g-extracted protein) = IC_50_ of gallic acid (mg/mL)/IC_50_ of extracted protein (g/mL) (3)

#### 2.5.2. 2,2′-Azino-bis(3-ethylbenzthiazoline-6-sulfonic acid) (ABTS) Radical Scavenging Assay

The ABTS radical scavenging assay was conducted using a modified procedure adapted from Lomakool et al. [18]. To prepare the ABTS radical cation, 0.0192 g of ABTS was dissolved in 5 mL of distilled water, while 0.3784 g of potassium persulfate was dissolved in 10 mL of water separately. The two solutions were combined in equal volumes and left to react in the dark for 16 h to form the ABTS•⁺ radical. Following incubation, the solution was diluted with distilled water until its absorbance reached 0.70 ± 0.02 at 734 nm. A standard curve was generated using Trolox for calibration. During the assay, 10 µL of the sample was added to 190 µL of the ABTS•⁺ solution in a 96-well microplate. After a 6 min incubation at room temperature, the absorbance was measured at 734 nm using a spectrophotometer. The antioxidant activity of the samples was expressed as Trolox equivalent (TE), calculated using the corresponding Equation (4).

ABTS activity (mg-TE/g-extracted protein) = IC_50_ of Trolox (mg/mL)/IC_50_ of extracted protein (g/mL) (4)

#### 2.5.3. Potassium-Ferricyanide-Reducing Antioxidant Power (PFRAP) Assay

Following the protocol described by Htoo et al. [15], a 0.2 M phosphate buffer at pH 6.6 was prepared. An amount of 30 milliliters of this buffer was combined with a 1% (*w*/*v*) potassium ferricyanide solution. Additionally, a 10% trichloroacetic acid (TCA) solution was prepared by dissolving 3 g of TCA in 30 mL of deionized water, while a 0.1% ferric chloride (FeCl_3_) solution was prepared by dissolving 0.03 g of FeCl_3_ in the same volume of water. A gallic acid standard curve was constructed for reference. In a 96-well plate, 145 µL of the phosphate buffer and 1% K_3_Fe(CN)_6_ were mixed with 60 µL of the sample and incubated at 50 °C for 20 min. Afterward, 145 µL of 10% TCA was added, and the mixture was centrifuged at 3000× *g* for 10 min. The resulting supernatant was combined with 500 µL of deionized water and 100 µL of 0.1% FeCl_3_. A control solution was prepared using 290 µL of phosphate buffer, 745 µL of deionized water, and 60 µL of the sample. The absorbance was measured at 700 nm, with higher absorbance values indicating greater reducing power. The PFRAP activity was expressed as mg-gallic acid equivalent (GAE)/g-extracted protein and calculated using Equation (5) below.

PFRAP activity (mg-GAE/g-extracted protein) = Absorbance at 1 of gallic acid/Absorbance at 1 of extract(5)

### 2.6. Anticancer Properties

#### 2.6.1. Cytotoxicity Assay Against Cancer Cells and Normal Cells

The cytotoxic activity of the extracts was evaluated using the MTT assay, based on the procedure outlined by Phinyo et al. [20]. Human colorectal adenocarcinoma cell lines (Caco-2 and HT-29) and normal Vero cells, obtained from SCB 2711 Microbiology Laboratory, Department of Biology, Faculty of Science, Chiang Mai University, Chiang Mai, Thailand, were cultured at 37 °C with 5% CO_2_ for 24 h. The culture medium for all cell types was Dulbecco’s Modified Eagle Medium (DMEM), supplemented with 10% heat-inactivated fetal bovine serum (FBS), penicillin (100 U/mL), and streptomycin (100 µg/mL). Additionally, DMEM containing pyruvate, 10% FBS, penicillin (100 U/mL), and streptomycin (100 µg/mL) was also used. Following the initial culture, approximately 2 × 10^5^ cells were seeded into 96-well plates and incubated under the same conditions for another 24 h. Cells were then treated with various concentrations of the extracts and incubated for an additional 48 h. Afterward, 30 µL of an MTT solution (2 mg/mL) was added to each well and incubated for 3 h to allow for formazan crystal formation. To dissolve the crystals, 200 µL of dimethyl sulfoxide (DMSO) was added. Absorbance readings were taken at 540 nm and 630 nm, and cell viability was calculated by comparing treated samples with untreated controls using the corresponding Equation (6).

Percentage of cell viability = [(A_0_ − A_1_)/A_0_] × 100% (6)

where A_0_ is the absorbance of the control and A_1_ is the absorbance of the extract.

#### 2.6.2. Measurement of the Cell Death by Flow Cytometry

To evaluate apoptotic cell death, flow cytometry with propidium iodide (PI) staining was performed following the method described by Kari et al. [21]. Cells were maintained at 37 °C in a 5% CO_2_ environment for 24 h before treatment with protein extracts to induce apoptosis. After treatment, cells were gently detached, collected into centrifuge tubes, and spun at 300× *g* for 5 min to form a pellet. The supernatant was discarded, and the cells were washed twice with phosphate-buffered saline (PBS) to remove any remaining cytotoxic substances. Subsequently, the cells were incubated with RNase (100 µg/mL) for 15 min to eliminate RNA contamination. After two more PBS washes, the cells were resuspended in PBS. Flow cytometry analysis was conducted using a CytoFlex LX system (Beckman Coulter, Brea, CA, USA) to determine the percentage of apoptotic cells. The percentage of apoptotic cells was calculated by comparing the treated samples to the untreated control using the corresponding Equation (7).

Apoptotic cells (%) = (No. of PI-positive cells/Total of cells) × 100%(7)

### 2.7. Functional Properties of Extracted Protein

#### 2.7.1. Solubility

To determine the solubility of the extracted protein, a modified version of the method described by Chaipoot et al. [22] was applied. A 0.5 g portion of the extracted protein was mixed with 50 mL of distilled water and vortexed for 3 min. Afterward, the mixture was centrifuged for 5 min, and the supernatant was carefully separated. The collected supernatant was transferred into pre-weighed moisture containers that had been pre-dried at 105 °C for 30 min and cooled in a desiccator. These containers were then placed in a drying oven at 105 °C and left overnight to ensure complete moisture removal. The protein solubility was calculated by measuring the weight of the dried residue using Equation (8) below.

Solubility (%) = (weight of supernatant after drying − weight of powder) × 100%(8)

#### 2.7.2. Foaming Capacity and Stability

The foaming characteristics of the extracted protein were evaluated following a modified method described by Rawdkuen et al. [23]. A 2 g sample of the protein was accurately measured and dissolved in 20 mL of distilled water using a graduated cylinder for precision. The mixture was then homogenized at 16,000 rpm for 2 min to promote dispersion and foam formation. To determine foaming capacity, the foam height was recorded immediately after homogenization (0 s) and after 30 s to detect any immediate reduction in foam volume. Foam stability was assessed by measuring the foam height at 0 s and again after 3 min to evaluate foam retention over time. The foaming capacity and stability were calculated using Equations (9) and (10), respectively, as follows.

Foaming capacity (%) = (total height at 30 s/total height at 0 s) × 100%(9)

Foaming stability (%) = (total height at 3 min/total height at 0 s) × 100%(10)

#### 2.7.3. Emulsifying Capacity and Stability

The emulsifying properties of the extracted protein were evaluated using a modified procedure based on Rawdkuen et al. [23]. To prepare for the emulsion, 10 mL of protein dispersion (prepared at a 1:10 weight-to-volume ratio) was combined with 10 mL of soybean oil. The mixture was homogenized at 27,000 rpm for 5 min to ensure uniform dispersion and emulsion formation. Immediately after homogenization, the total emulsion height was recorded to assess the sample’s emulsifying capacity, indicating its ability to stabilize the oil–water interface.

To test emulsion stability, the prepared emulsion was transferred into a water bath and heated at 80 °C for 30 min. After heating, the emulsion height was measured again to evaluate the stability of the emulsion under thermal conditions. The change in emulsion height before and after heating indicated the sample’s resistance to structural breakdown. Emulsifying capacity and stability were calculated using Equations (11) and (12), respectively, as follows.

Emulsifying capacity (%) = (total height after homogenization/initial height) × 100%(11)

Emulsifying stability (%) = (total height after heating/initial height) × 100%(12)

#### 2.7.4. Digestibility

Protein dispersion was prepared by dissolving the sample in distilled water and adding pepsin at an enzyme-to-protein ratio of 1:100 (*w*/*w*), following a modified method based on Sharma et al. [6]. The pH of the mixture was adjusted to 2 using 0.5 mol/L HCl, and the reaction was conducted at 37 °C for 120 min with gentle stirring to facilitate pepsin digestion. Enzymatic activity was terminated by placing the reaction mixture in boiling water for 10 min. After cooling, the pH was neutralized using 1.0 mol/L NaOH to deactivate any remaining pepsin. Trypsin was then introduced at the same enzyme-to-protein ratio of 1:100 (*w*/*w*). The trypsin digestion was performed under identical conditions, with the temperature maintained at 37 °C and samples collected at intervals over a 240 min period. The trypsin digestion process was halted after 120 min by boiling the mixture for 10 min. The in vitro protein digestibility (%) of the digested samples was assessed by measuring protein content using the Lowry method [14], with bovine serum albumin serving as the standard. Digestibility was calculated by comparing the total soluble protein content before and after digestion, as indicated by the following Equation (13).

Protein digestibility (%) = 100% − [(Pd/Pt) × 100%](13)

where Pt is the total protein content before digestion, and Pd is the protein content remaining after in vitro digestion.

### 2.8. Statistical Analysis

Results are presented as the mean ± standard deviation (SD) based on three separate experiments. Statistical evaluation was conducted using one-way analysis of variance (ANOVA) with SPSS software version 20 (IBM, Armonk, NY, USA). Differences were deemed statistically significant when the *p*-value was less than 0.05.

## 3. Results and Discussion

### 3.1. Optimization of Ultrasound-Assisted Deep Eutectic Solvent Extraction of Protein from Spirulina Biomass

#### 3.1.1. Statistical Analysis

A statistical approach was employed to model protein extraction from *Spirulina* biomass using deep eutectic solvents (DESs), utilizing the central composite design (CCD) within the framework of response surface methodology (RSM). Upon completing the experimental trials, the findings are systematically presented in Table 1. A second-order quadratic polynomial regression model was formulated to define the correlation between the input factors and the extraction yield. The developed statistical model for protein recovery is expressed in the following Equation (14) below, where the independent variables—biomass loading, sonication amplitude, and extraction duration—are represented in coded terms to conform to the quadratic model structure. For clarity, biomass (%), amplitude (%), and reaction time (minutes) are denoted as A, B, and C, respectively.

Protein yield (%) = 74.90 + 5.55A − 2.68B + 0.38C − 5.09 × 10^−2^AB − 4.90 × 10^−2^AC + 1.40 × 10^−3^BC − 5.18 × 10^−1^A^2^ + 5.46 × 10^−2^B^2^ − 1.63 × 10^−4^C^2^ (14)

The second-order polynomial equations derived characterize the empirical relationships between independent factors and the protein extraction yield. In these equations, positive coefficients signify a beneficial effect, indicating an increase in extraction efficiency, while negative coefficients denote inhibitory influences, resulting in a reduction in yield. To evaluate the reliability and predictive accuracy of the developed quadratic models, an analysis of variance (ANOVA) was conducted. The statistical significance and adequacy of these models are detailed in Table 2. ANOVA results demonstrate the statistical significance and adequacy of the quadratic model in describing protein extraction from *Spirulina* biomass using deep eutectic solvents. The model exhibits a high level of significance, with an F-value of 106.28 and a *p*-value of <0.0001, indicating that the selected factors significantly influence the extraction yield. Among the individual parameters, biomass (A), sonication amplitude (B), and sonication time (C) all exhibit highly significant effects on protein extraction yield (*p* < 0.0001). Sonication amplitude (B) shows the strongest impact, with the highest sum of squares (552.51) and an F-value of 541.02, indicating that variations in amplitude contribute most to yield differences. Biomass concentration (A) and sonication time (C) also significantly influence extraction, with F-values of 213.81 and 204.58, respectively. The interaction terms AB (biomass concentration × amplitude) and AC (biomass concentration × sonication time) are statistically significant (*p* < 0.05), suggesting that these factor combinations have a synergistic effect on the extraction yield. In contrast, the interaction BC (amplitude × sonication time) is not significant (*p* = 0.5801), indicating that changes in amplitude and sonication time do not interact in a way that substantially affects the yield. The residual sum of squares is 5.11, with a non-significant lack of fit (*p* = 0.3403), confirming that the model adequately represents the data without systematic errors [24]. The model achieves an R^2^ value of 0.9948, indicating that 99.48% of the variation in protein extraction yield is explained by the model. This high R^2^ value demonstrates the strong predictive capability of the quadratic model. The model’s adequate precision value of 40.882 suggests a strong signal-to-noise ratio, reinforcing the model’s reliability for predictive purposes. The statistical analysis confirms that the quadratic model is well fitted and highly predictive of protein extraction yield. Sonication amplitude (B) emerges as the most influential factor, followed by biomass concentration (A) and sonication time (C). The significant interaction effects of AB and AC highlight the importance of optimizing these factor combinations. The non-significant term (BC) suggests that some interactions and quadratic effects may have negligible influence on extraction yield. Overall, the model provides a robust framework for optimizing extraction conditions and maximizing protein yield.

#### 3.1.2. Effect of Parameters on Protein Yield

**Figure 1:**
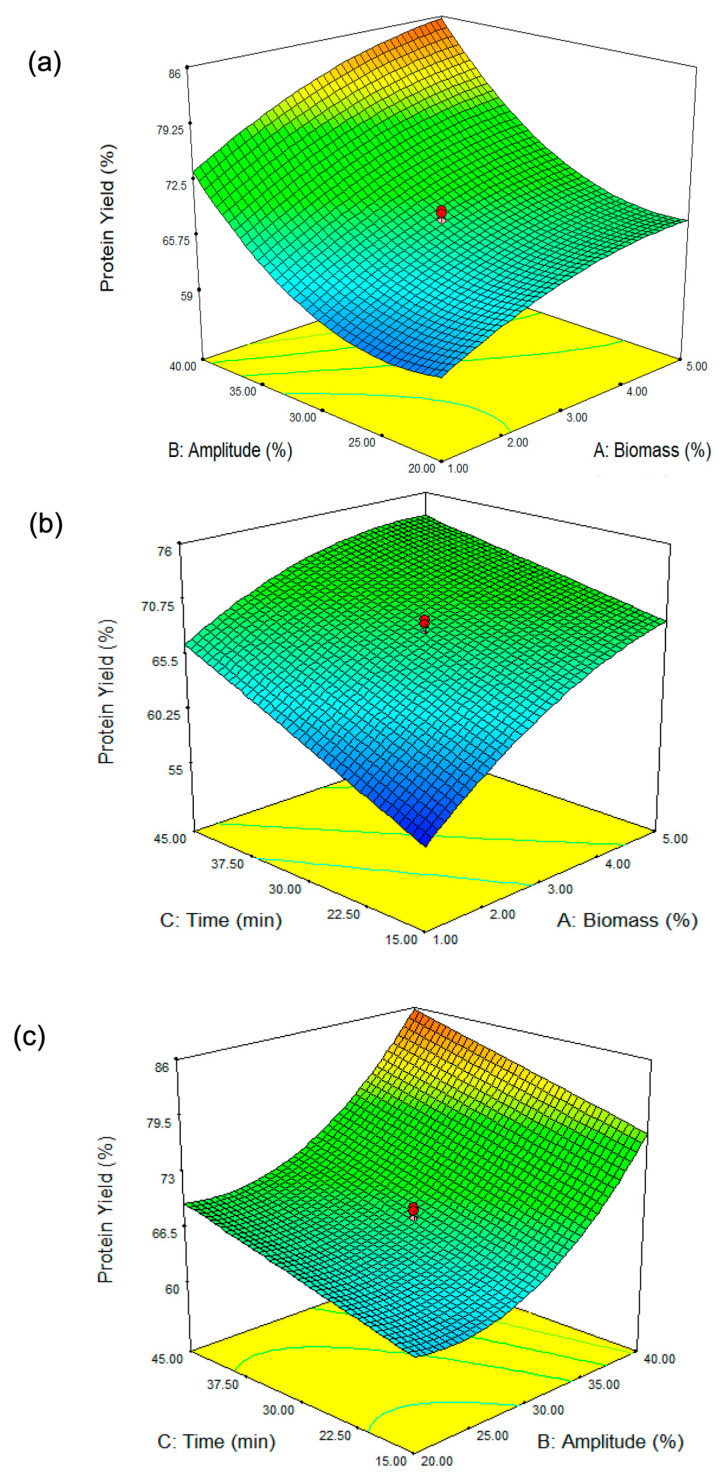
Three-dimensional surface plots showing the influence of (**a**) biomass concentration and amplitude, (**b**) biomass concentration and sonication time, and (**c**) amplitude and sonication time on protein yield from ultrasound-assisted DES extraction of *Spirulina* biomass. The color gradient (from green to orange) represents the variation in protein yield, with green indicating lower yields and orange indicating higher yields. The green contour lines highlight specific parameter thresholds, while the red dots correspond to the experimental data points, with the optimized conditions marked for reference.

From Figure 1a, it is evident that sonication amplitude (B) has a stronger influence on protein yield compared to biomass concentration (A). At low biomass concentrations, increasing amplitude significantly enhances protein yield, as indicated by the steeper slope of the surface. This suggests that when biomass content is low, ultrasonic energy is efficiently transmitted through the extraction medium, resulting in effective cell disruption and higher protein release. Sonication amplitude, on the other hand, is a critical parameter governing the intensity of ultrasonic energy applied to the system. At lower amplitude levels (~20–25%), protein yield remains relatively low across different biomass concentrations. This suggests that the applied ultrasonic energy is insufficient to effectively disrupt the *Spirulina* cell walls, leading to limited protein release. As amplitude increases towards 35–40%, a noticeable rise in protein yield is observed, demonstrating the crucial role of ultrasonic intensity in breaking down the cell structure and improving mass transfer. This finding aligns with that of Kingwascharapong et al. [25], who observed a similar trend, highlighting the impact of increasing sonication amplitude on enhancing protein yield from the Bombay locust. At lower amplitudes, the cavitation effect is weaker, leading to insufficient cell disruption and lower protein yield. As the amplitude increases, cavitation becomes more intense [26], effectively breaking down *Spirulina* cell walls and releasing intracellular proteins into the solvent. However, excessive amplitude may generate excess heat, potentially causing protein denaturation and reducing overall yield. At higher biomass concentrations in our study, the increase in amplitude shows a diminished effect on protein yield. At higher biomass concentrations, the increased viscosity and reduced solvent availability can hinder ultrasonic wave propagation, limiting cavitation efficiency and reducing protein extraction [27]. The curvature of the surface flattens in this region, indicating that beyond a certain point, increasing sonication amplitude does not lead to a proportional increase in protein extraction. This could be attributed to higher viscosity and limited cavitation efficiency, which reduce the penetration of ultrasonic waves and hinder mass transfer. The interaction effect (AB) is evident in the way the surface bends: at low biomass concentrations, increasing amplitude has a strong positive effect, while at high biomass concentrations, this effect weakens. This interaction suggests that optimizing amplitude is more critical when working with lower biomass concentrations, whereas at higher biomass levels, other factors such as sonication duration or solvent properties might play a more dominant role in determining protein yield.

The response surface plot (Figure 1b) illustrates the interaction between biomass concentration (A, %) and extraction time (C, min) on *Spirulina* protein yield (%). The results indicate that at lower biomass concentrations (approximately 1%), protein yield remains relatively low, while an increase in biomass concentration leads to an improvement in yield. However, this trend begins to plateau at higher biomass levels (4–5%), suggesting diminishing returns. Similarly, extraction time plays a crucial role in protein yield, with shorter durations (around 15 min) resulting in lower yields, whereas extending the extraction time enhances protein yield, reaching an optimum at approximately 45 min. This finding aligns with the study by Wen et al. [28], which reported that extending ultrasonic treatment duration led to increased protein yields in *Porphyra haitanensis* (a red alga). Beyond this point of 45 min in our study, further prolongation does not appear to significantly increase yield. The interaction between these two parameters is evident in the curvature of the response surface, indicating that neither biomass concentration nor extraction time alone is solely responsible for maximizing protein yield. Instead, the highest protein yield is achieved when both biomass concentration and extraction time are optimized together, with moderate biomass levels (~3–4%) and longer extraction times (~40–45 min) providing the most favorable conditions. This finding highlights the importance of balancing these factors to achieve efficient protein extraction from *Spirulina* biomass.

Figure 1c illustrates the combined effect of amplitude (B, %) and extraction time (C, min) on protein yield (%). The results indicate that both parameters significantly influence protein recovery. At lower amplitude levels (approximately 20%), the protein yield remains relatively low regardless of extraction time. As the amplitude increases, protein yields improve substantially, particularly when combined with extended extraction times. The curvature of the response surface suggests that higher amplitude settings (above 35%) and longer extraction durations (above 40 min) maximize protein extraction efficiency. However, the ANOVA results (Table 2) indicate that the interaction between amplitude (B, %) and extraction time (C, min) is non-significant, suggesting that while both factors may individually influence protein yield, their combined effect does not create a statistically meaningful interaction. Despite the visual trend observed in the response surface plot, the lack of statistical significance means that changes in one factor do not strongly depend on the other in determining protein yield. Thus, while increasing amplitude and extraction time individually contributes to higher protein yield, their interaction does not have a synergistic or antagonistic effect. This finding implies that optimizing each parameter independently may be more relevant than focusing on their combined influence.

#### 3.1.3. Numerical Optimization and Verification of the Model

The optimization process determined the ideal conditions for maximizing protein yield. The optimal parameters consisted of a biomass concentration of 5%, an amplitude of 40%, and a sonication duration of 22.14 min. Based on these conditions, the predicted protein yield was 84.27%. To assess the accuracy of the model, experimental validation was conducted using 5% biomass, 40% amplitude, and a sonication time of 22 min, resulting in an actual protein yield of 80.62%. The discrepancy between the predicted and experimental values was calculated to be 4.33%, demonstrating a strong correlation between the model’s predictions and the experimental outcomes. This minimal deviation highlights the model’s effectiveness in accurately optimizing protein extraction parameters.

A prior study reported that ultrasound-assisted alkaline extraction (UAAE) of *Arthrospira platensis* (formerly known as *Spirulina platensis*) protein at a 60% amplitude level and 30 min sonication time using 4 g of biomass at pH 12 resulted in the highest protein yield of 81.86% [12]. In comparison, our study demonstrated that using a 40% amplitude and a sonication time of 22 min, with choline chloride–urea (ChCl:Urea) as the extraction solvent and 5 g of biomass at the same pH 12, resulted in a protein yield of 80.62%. This yield is only slightly lower than the previously reported value despite using a lower amplitude, shorter extraction time, a different extraction solvent, and a higher biomass load. The differences in amplitude, sonication time, extraction solvent, and biomass amount between the two studies suggest that lower intensity and reduced exposure duration may still yield a comparably high protein recovery. A 60% amplitude level leads to stronger cavitation effects, potentially causing more effective cell disruption, but prolonged exposure may induce protein degradation due to excessive energy input. Additionally, in our previous study, the use of an alkaline solvent for *Spirulina* biomass extraction resulted in a lower protein yield of 11.84% [19], emphasizing the superior efficiency of the DES-based ultrasound-assisted extraction method. In contrast, our study optimized conditions using a deep eutectic solvent (ChCl:Urea), which provided sufficient disruption while minimizing possible degradation, thereby maintaining high protein yield. When considering extraction efficiency, it is important to assess both yield and process optimization. Braspaiboon et al. [12] achieved a marginally higher yield (81.86%) with a higher amplitude (60%) and longer sonication time (30 min). However, our study demonstrated a nearly comparable yield (80.62%) with reduced energy input (40% amplitude, 22 min) and a different solvent system (ChCl: Urea), which may offer advantages in terms of sustainability and protein stability.

Another study achieved a protein extraction yield of 40% by dispersing *Spirulina* biomass in ultra-purified water at a concentration of 30 g/L and adjusting the pH to 9 using a 2 mol/L NaOH solution [29]. The sample was then sonicated in an ultrasound bath for 45 min at 30 °C, with the goal of enhancing protein release through cell wall disruption. Compared to this method, our study achieved a significantly higher protein yield of 80.62%, despite using a shorter sonication time (22 min) and a different extraction solvent (ChCl:Urea) instead of ultra-purified water. This suggests that our approach is more efficient in enhancing protein recovery, likely due to the effectiveness of deep eutectic solvents in improving cell wall disruption and protein solubilization. The extraction conditions (300 W sonication power, 10 min total duration) in the study of Guo et al. [30] yielded a protein extraction efficiency of 25%, whereas in our study, response surface methodology (RSM) using a central composite design (CCD) optimized the ultrasound-assisted extraction (UAE) conditions, achieving a significantly higher yield of 80.62%. The key differences contributing to this improvement include ultrasound parameters, extraction duration, and solvent selection. The reference study employed a direct sonication power of 300 W, while our study optimized 40% amplitude and 22 min of sonication, ensuring more effective cell disruption and protein release. Additionally, the use of deep eutectic solvent (DES) (ChCl:Urea) in our study likely enhanced protein solubility and extraction efficiency, in contrast to conventional extraction solvents. The extended sonication duration, combined with DES, facilitated greater protein recovery. The use of RSM-CCD allowed precise optimization of these parameters, leading to a significantly higher yield. This comparison highlights the advantages of statistical optimization and green solvent integration in maximizing protein extraction efficiency from *Spirulina* biomass. Overall, while each method in previous studies is effective, our optimized UAE method demonstrates an efficient alternative that balances protein yield with reduced energy consumption and potential improvements in sustainability. Additionally, although RSM was used to optimize the extraction process, further fine-tuning of parameters may yield additional improvements in protein recovery and bioactivity. Incorporating other optimization techniques, such as artificial intelligence-driven modeling, could provide deeper insights into process refinement.

### 3.2. Characterization of Protein-Rich Extracts from Spirulina Biomass

#### 3.2.1. Protein Content and Impurities

Table 3 presents the composition of protein-rich samples extracted from *Spirulina* biomass using DES integrated with ultrasonication. The extracted protein concentration was 442.88 mg/g extract, indicating a highly efficient extraction process. The use of DES combined with ultrasonication likely enhanced protein solubilization, leading to a high yield, making this method promising for protein. The extraction of protein from *Spirulina* using the methods outlined in this study resulted in a concentration of 44.29%, which, while significant, falls short of the remarkable maximum concentration of 66.6% achievable through optimized extraction techniques as reported in the study of Silva et al. [29]. This discrepancy highlights the importance of methodical optimization in protein extraction processes, as various parameters such as amplitude, sonication time, and biomass concentration can substantially influence yield. The lower concentration achieved in this study could be attributed to the specific conditions employed, including a more moderate sonication amplitude of 40% and a shorter sonication duration of 22 min. Previous studies have indicated that higher ultrasonic frequencies and extended exposure times can enhance cavitation, leading to more effective disruption of cell walls and improved protein solubilization. The protein content of the *Spirulina* extract in our study (44.29%) is slightly lower than that of animal-based proteins, such as egg protein (51%) [31]. However, it remains a highly valuable source of plant-based protein, especially considering its sustainability, bioactivity, and ease of production. While animal proteins generally provide a complete amino acid profile, *Spirulina* protein is also considered highly digestible and rich in essential amino acids, making it a strong alternative for plant-based diets. Additionally, *Spirulina* contains bioactive compounds, such as antioxidants, which offer health benefits beyond just protein nutrition. Unlike egg protein, which is limited by production scalability and ethical concerns, *Spirulina* can be cultivated efficiently with lower environmental impact, requiring less water and land compared to livestock production.

During protein extraction, the simultaneous extraction of undesirable compounds as interferents, including sugars and phenolic substances, can occur [32]. The total phenolic content was 4.87 mg GAE/g extract, suggesting the presence of bioactive compounds that may contribute to antioxidant properties, although the concentration is relatively low compared to dedicated phenolic extractions. Additionally, the total sugar content was 0.18 mg/mL, indicating minimal co-extraction of carbohydrates. This suggests that the applied extraction method effectively targeted proteins while limiting sugar contamination, which is advantageous for purity in protein applications. Overall, these results highlight the effectiveness of DES-ultrasonication for obtaining protein-rich extracts with bioactive potential, supporting its application in functional food or nutraceutical formulations.

#### 3.2.2. Amino Acids Composition by HPLC Analysis

The nutritional quality of dietary proteins depends on the amount and bioavailability of essential amino acids (EAAs), which are vital for growth, longevity, and overall metabolic function in humans. Proteins derived from plant sources often lack adequate levels of one or more EAAs, such as branched-chain amino acids, lysine, methionine, or tryptophan, which can limit their effectiveness as a sole protein source [33]. The amino acid composition of the *Spirulina* protein extract (Table 4) highlights its nutritional value and limitations in meeting essential amino acid requirements. The extract contains 39.91% essential amino acids (EAAs) relative to the total amino acid content, which take up beyond a third of total amino acid composition, indicating its potential as a valuable protein source. Our study can be compared to the EAAs (39.42%) relative to the total amino acids of *Spirulina* extracted by microwave-assisted extraction in the report of Zin et al. [19]. This similarity suggests that ultrasound-assisted extraction with DES is an effective alternative for preserving essential amino acids in *Spirulina* protein. Notably, leucine (7.32 g/100 g) exceeds the recommended intake for both children and adults, supporting its role in muscle synthesis and metabolic regulation. Leucine is a potent activator of the mechanistic target of rapamycin complex 1 (mTORC1), which is crucial for protein synthesis. When leucine levels are elevated, it stimulates the mTORC1 pathway, enhancing protein translation and ribosome biogenesis, leading to increased muscle protein synthesis (MPS) [34]. However, other essential amino acids, such as threonine (1.92 g/100 g), valine (2.46 g/100 g), and isoleucine (2.52 g/100 g), are slightly below the WHO/FAO/UNU recommendations, which may require dietary supplementation for optimal protein quality.

More critically, methionine (0.36 g/100 g) and lysine (0.33 g/100 g) are significantly lower than the recommended levels, making *Spirulina* an incomplete protein source if consumed alone. Beyond essential amino acids, the extract is rich in acidic amino acids (29.27%), including aspartic acid and glutamic acid, which contribute to umami flavor [35]. Additionally, 50.11% of the total amino acids are hydrophobic, influencing protein stability, structure, and potential applications in food formulations. The presence of arginine (3.70 g/100 g), a basic amino acid, further enhances its value due to its role in immune function and cardiovascular health. L-arginine supplementation can boost the production of key cytokines like interleukin-1 beta and TNF-alpha, which play vital roles in the innate immune response. It supports the integrity of the endothelial lining of blood vessels, ensuring proper vascular function and preventing endothelial dysfunction, which is a significant contributor to cardiovascular diseases [36]. While *Spirulina* protein extract is a high-quality plant protein, its deficiency in lysine and methionine suggests that it should be combined with complementary protein sources (such as legumes or cereals) to achieve a balanced amino acid profile.

#### 3.2.3. Molecular Weight Determination by SDS-PAGE

**Figure 2:**
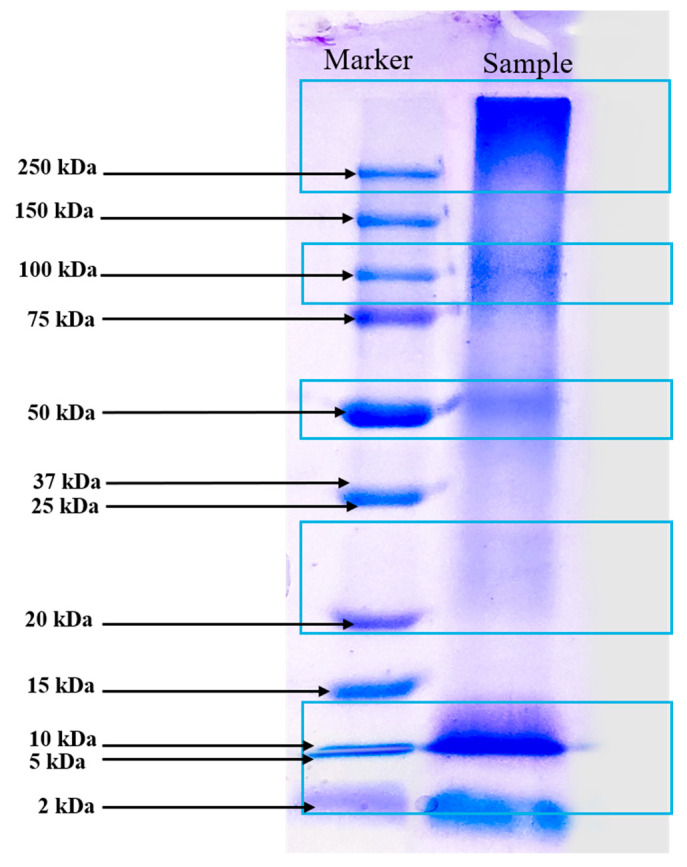
Molecular pattern of extracted *Spirulina* protein by SDS-PAGE analysis (Marker displays Precision Plus Protein™ Dual Color Standards markers ranging from 2 kDa to 250 kDa).

The SDS-PAGE analysis of the protein-rich extract from *Spirulina* biomass revealed distinct protein bands at approximately 10 kDa, 20–25 kDa, 50 kDa, 100 kDa, and >250 kDa, reflecting a diverse protein composition (Figure 2). The presence of low-molecular-weight proteins (10 kDa and 20–25 kDa) suggests the existence of small bioactive peptides or enzymatic proteins, possibly including phycobiliproteins such as phycocyanin subunits, which are known for their antioxidant and immunomodulatory properties. Julianti et al. [37] stated in a similar study that protein bands between 12 and 21 kDa are consistent with the α and β subunits of C-phycocyanin. The α and β subunits of C-phycocyanin are protein components that, when combined, form the functional C-phycocyanin molecule, a blue pigment primarily found in cyanobacteria. Their primary function is to capture light energy during photosynthesis, transferring it to chlorophyll, while also exhibiting various bioactive properties like antioxidant, anti-inflammatory, and potential anticancer effects due to their unique structure and amino acid composition [38]. The presence of proteins at 50 kDa and 10 kDa could indicate the large (L) and small (S) subunits of RuBisCO, an essential enzyme for carbon fixation in autotrophic microorganisms [39]. The 100 kDa band likely corresponds to microalgae protein-membrane components, as reported by Rajakumar and Muthukumar [40]. Additionally, the high-molecular-weight proteins exceeding 250 kDa could represent large protein complexes or aggregated forms. DES-based extraction reported in our study improved protein extraction efficiency by disrupting the cell wall structure and facilitating the release of intracellular proteins while maintaining bioactivity. The integration of UAE further enhances extraction efficiency by promoting cavitation, which aids in breaking down protein aggregates and improving yield. This method likely contributed to the diverse range of proteins observed in the SDS-PAGE profile. The broad molecular weight distribution observed in the extract highlights the potential functional and bioactive applications of *Spirulina*-derived proteins, ranging from antioxidant and anti-inflammatory effects to enzymatic and structural roles. However, future research could investigate chromatography-based methods to obtain a more detailed molecular weight distribution analysis.

### 3.3. Antioxidant Activities

**Figure 3:**
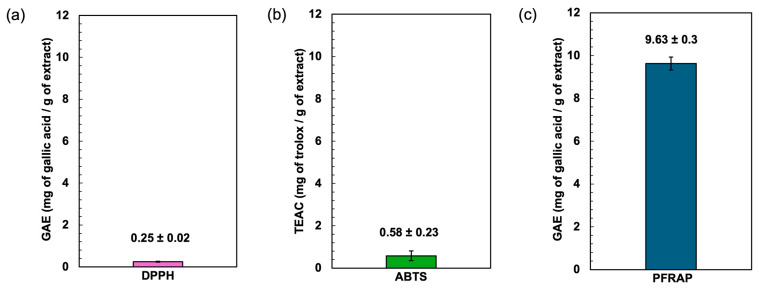
Effect of antioxidants determined by DPPH radical scavenging assay (**a**), ABTS radical scavenging assay (**b**), and potassium ferricyanide-reducing antioxidant power (PFRAP) (**c**).

The antioxidant potential of the *Spirulina* protein-rich extract was assessed using DPPH, ABTS, and ferric reducing power (PFRAP) assays. The results demonstrated the extract’s ability to neutralize free radicals and reduce ferric ions, indicating its potential as a natural antioxidant source (Figure 3). The DPPH radical scavenging assay is a widely used method for evaluating the ability of compounds to donate hydrogen atoms or electrons, neutralizing the DPPH radical. The *Spirulina* protein-rich extract exhibited a DPPH radical scavenging activity of gallic acid equivalent to 0.25 mg of gallic acid/g of extract, suggesting a moderate capacity to scavenge free radicals. Aromatic amino acids such as tyrosine (Tyr) possess excellent hydrogen-donating abilities and demonstrate significant radical-scavenging properties [41]. The relatively low DPPH activity compared to standard antioxidants (gallic acid) may be attributed to the nature of proteins, which primarily function as metal ion chelators and radical quenchers rather than direct hydrogen donors. This characteristic may be influenced by the presence of basic and acidic amino acids such as arginine, lysine, histidine, aspartic acid, and glutamic acid, which have the potential to chelate metal ions through their side-chain carbonyl and amino groups [42].

The ABTS radical cation decolorization assay measures the ability of antioxidants to quench ABTS radicals. The assay is based on the generation of a blue/green ABTS^+^, which is suitable for detecting both hydrophilic and hydrophobic antioxidant compounds. In contrast, the DPPH assay is stable and generates radicals that can be dissolved in organic solvents, making it applicable for detecting hydrophobic compounds [43,44]. The *Spirulina* protein-rich extract showed an ABTS radical scavenging activity of 0.58 mg of trolox/g of extract, indicating a higher radical scavenging capacity compared to the DPPH assay. This result suggests that the extract contains bioactive compounds, such as peptides or amino acids, that exhibit stronger interactions with ABTS radicals. The higher activity in the ABTS assay may also be due to the extract’s ability to stabilize radical cations more effectively than the neutral DPPH radical. Trolox was used as the standard for the ABTS assay, and the results suggest the extract’s antioxidant potential relative to this benchmark compound.

The ferric reducing antioxidant power (PFRAP) assay evaluates the extract’s capacity to reduce Fe^3^⁺ to Fe^2^⁺, reflecting electron-donating potential. This reducing power is crucial in mitigating oxidative stress by breaking free radical chain reactions and preventing the oxidation of biomolecules. The *Spirulina* protein-rich extract exhibited a gallic acid equivalent of 9.63, demonstrating a strong reducing ability. The antioxidant compounds present in phycocyanin contribute to the reduction of Fe^3+^ to Fe^2+^ [45]. The result implies that the extract contains bioactive compounds capable of transferring electrons, which could contribute to its overall antioxidant properties. Overall, the results suggest that the *Spirulina* protein-rich extract possesses notable antioxidant activity, with a particularly strong ferric ion reducing power. The differences in activity observed across the three assays may be attributed to variations in the mechanisms of action, solubility, and interactions of the bioactive compounds with different radical species. Further studies on peptide characterization and structure–activity relationships could provide deeper insights into the antioxidant mechanisms of *Spirulina*-derived proteins.

### 3.4. Anticancer Activity

**Figure 4:**
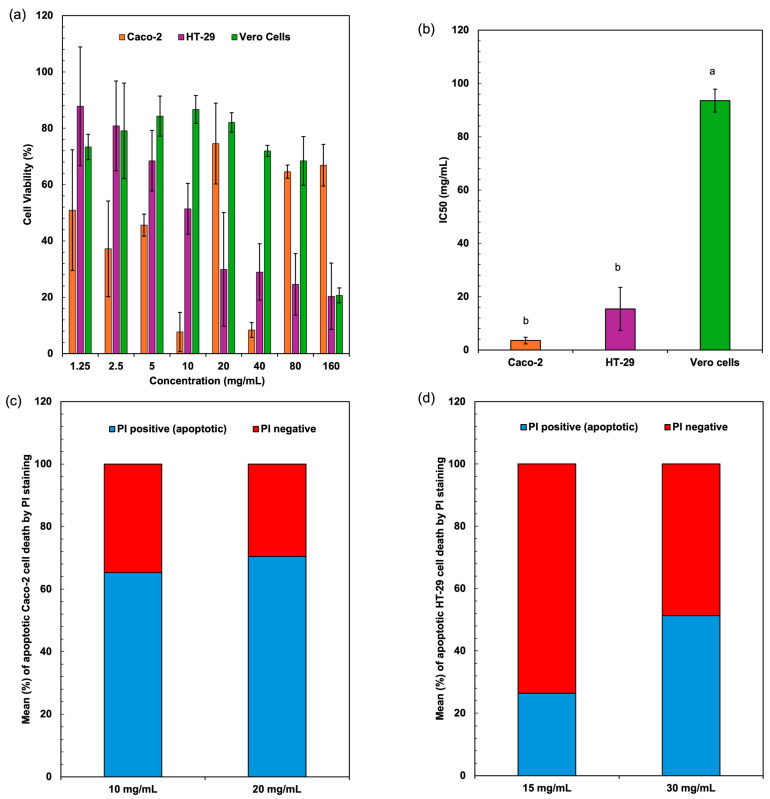
Cell viability values (**a**) and cytotoxicity IC_50_ values (**b**) of colon cancer cell lines (Caco-2, HT-29) and normal (Vero) cells after treatment with extracted proteins from *Spirulina* biomass. Apoptotic cell death assessed by flow cytometry using PI staining for Caco-2 (**c**) and HT-29 (**d**) cells. Different lowercase letters (a, b) indicate significant differences among the treatments (*p* < 0.05).

Figure 4a represents the cell viability (%) of three different cell lines Caco-2, HT-29, and Vero cells at various concentrations (mg/mL) of *Spirulina* protein. The cytotoxic effects of *Spirulina* protein on Caco-2, HT-29, and Vero cells demonstrate a dose-dependent trend, with a significant reduction in cell viability observed in cancerous cell lines (Caco-2 and HT-29) compared to non-cancerous Vero cells. At lower concentrations (1.25–10 mg/mL), both Caco-2 and HT-29 cells exhibit moderate viability reductions, whereas higher concentrations (40–160 mg/mL) cause a more pronounced cytotoxic effect, with HT-29 cells showing the lowest viability. In contrast, Vero cells maintain relatively high viability across all concentrations, suggesting that *Spirulina* protein selectively affects cancer cells while sparing normal cells. Figure 4b presents the IC_50_ values (mg/mL) of the extracts against three different cell lines. The IC_50_ value (half-maximal inhibitory concentration) indicates the concentration required to reduce cell viability by 50%. Lower IC_50_ values suggest higher cytotoxicity, whereas higher IC_50_ values indicate lower cytotoxicity. The extract shows strong cytotoxic effects against colorectal cancer cells (Caco-2 and HT-29), particularly Caco-2, which had the lowest IC_50_. The significantly higher IC_50_ for Vero cells suggests that the sample has low toxicity towards normal cells. This selective cytotoxicity is a promising indicator of *Spirulina* protein’s potential as an anticancer agent. Shaik and Harikrishnan [46] demonstrated that phycocyanin from *Spirulina* exerted strong cytotoxicity against HT-29 cells (IC_50_ = 23.30 µg/mL) while sparing Vero cells (IC_50_ = 152.2 µg/mL), aligning with the selective cytotoxicity trend observed in this study.

The underlying mechanisms responsible for this cytotoxicity may involve several biochemical pathways. One possible mechanism is the induction of apoptosis in cancer cells through the generation of reactive oxygen species (ROS). *Spirulina*-derived proteins and peptides have been reported to exhibit antioxidant and pro-oxidant activities, which can trigger oxidative stress in cancer cells, leading to mitochondrial dysfunction and apoptosis. Additionally, bioactive peptides from *Spirulina* may interact with cell surface receptors, initiating apoptotic signaling cascades such as the intrinsic (mitochondria-mediated) and extrinsic (death receptor-mediated) pathways [47]. The observed higher cytotoxicity in HT-29 cells compared to Caco-2 cells suggests that different colorectal cancer cell lines may exhibit varying sensitivities to *Spirulina* protein, possibly due to differences in their metabolic activities, receptor expression, or intracellular antioxidant defense mechanisms. Another potential mechanism involves cell cycle arrest, where bioactive compounds in *Spirulina* protein interfere with key regulators of cell proliferation, such as cyclins and cyclin-dependent kinases (CDKs) [48]. By inhibiting these regulators, *Spirulina* protein may prevent cancer cells from progressing through the cell cycle, ultimately leading to growth inhibition and cell death. Furthermore, *Spirulina*-derived peptides may exert cytotoxic effects by modulating key signaling pathways involved in cancer progression, such as the PI3K/Akt and MAPK pathways, which regulate cell survival and proliferation [49,50]. Inhibition of these pathways could enhance cancer cell sensitivity to apoptosis while maintaining the viability of normal cells like Vero cells.

The significantly higher cell viability of Vero cells across all tested concentrations suggests that *Spirulina* protein may preferentially target cancer cells while exerting minimal toxicity on normal cells. This selectivity is critical for potential therapeutic applications, as it reduces the risk of adverse effects commonly associated with conventional chemotherapy. A study on *Spirulina platensis* methanol extract encapsulation reported similar findings, where cytotoxic effects were observed in Caco-2 and HepG-2 cells but not in Vero cells, further supporting the safety of *Spirulina* protein for normal cells [51], which supports our findings on Caco-2 cell sensitivity. The ability of *Spirulina* protein to differentiate between cancerous and normal cells may be attributed to differences in metabolic activity, membrane composition, or specific receptor interactions that make cancer cells more susceptible to its bioactive components. Figure 4b presents the IC_50_ values, indicating the concentration required to reduce cell viability by 50%. The lower IC_50_ values for Caco-2 and HT-29 cells confirm the potent anticancer effect of *Spirulina* protein, whereas the significantly higher IC_50_ for Vero cells suggests resilience against cytotoxic effects. This selectivity is crucial for therapeutic applications, minimizing toxicity to normal tissues while effectively inhibiting cancer cell growth. The differential response may be linked to variations in metabolic stress tolerance, membrane composition, or specific receptor interactions between cancerous and normal cells.

**Figure 5:**
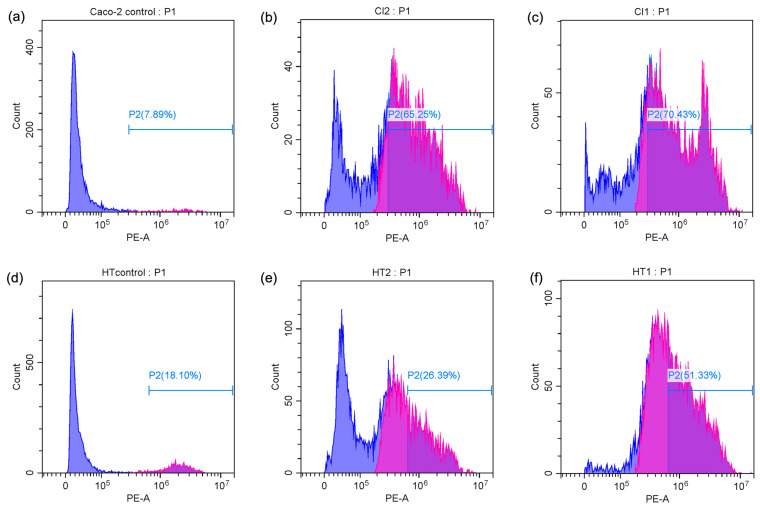
Flow cytometry analysis of PI content in apoptotic Caco-2 cells ((**a**) control cells, (**b**) treatment with 10 mg/mL *Spirulina* protein, and (**c**) treatment with 20 mg/mL *Spirulina* protein) and apoptotic HT-29 cells ((**d**) control cells, (**e**) treatment with 15 mg/mL *Spirulina* protein, and (**f**) treatment with 30 mg/mL *Spirulina* protein). Blue color stands for PI positive and purple color represents PI negative (Apoptotic cells).

The percentage of apoptotic cells was significantly elevated in both Caco-2 and HT-29 cell lines when treated with *Spirulina* protein at varying concentrations. Figure 4c,d present the average apoptotic cell percentages for the Caco-2 and HT-29 cell lines. Figure 5 shows the cell response detected by 24 h PI staining, which allows for the detection of apoptotic cells by identifying DNA fragmentation, revealing that in Caco-2 cells, the apoptotic rate was 65.25% at 10 mg/mL and 70.43% at 20 mg/mL, indicating a dose-dependent induction of apoptosis. Similarly, in HT-29 cells, the apoptotic rate at 15 mg/mL was 26.39%, which increased to 51.33% at 30 mg/mL. This also indicates a dose-dependent induction of apoptosis, with a more substantial increase in apoptosis observed at the higher concentration of *Spirulina* protein. The present study demonstrates that *Spirulina* protein induces a potent apoptotic response in both Caco-2 and HT-29 colon cancer cell lines, as evidenced by propidium iodide (PI) staining. PI staining is a reliable method for detecting late-stage apoptosis, as it binds to DNA fragments in dying cells, allowing for quantification of apoptotic cell death [52]. In Caco-2 cells, treatment with *Spirulina* protein at 10 mg/mL and 20 mg/mL resulted in substantial apoptosis, with a slightly higher apoptotic rate observed at the 20 mg/mL concentration. This suggests that the apoptotic effect of *Spirulina* protein is concentration-dependent, with 20 mg/mL potentially representing an optimal dose for inducing apoptosis in Caco-2 cells.

In HT-29 cells, the apoptotic response was more markedly enhanced at higher concentration, with a notable increase in apoptosis from 26.39% at 15 mg/mL to 51.33% at 30 mg/mL. This larger increase in apoptosis at higher concentrations suggests that HT-29 cells may exhibit greater sensitivity to *Spirulina* protein, or the protein may exert a more pronounced effect at higher concentrations. The observed dose-dependent effect in both cell lines underscores the potential for *Spirulina* protein as a therapeutic agent in colon cancer treatment. These results align with the growing body of evidence supporting the anticancer properties of *Spirulina* and its bioactive components, suggesting that it may play a role in promoting programmed cell death in cancer cells. A study has demonstrated that C-phycocyanin (C-PC), a component of *Spirulina platensis*, induces apoptosis in MCF-7 breast cancer cells by elevating the levels of key caspases involved in apoptotic pathways. Specifically, caspase 9 was highlighted for its role in the intrinsic apoptotic pathway, while caspase 8 was associated with the extrinsic pathway, indicating that both pathways were modulated by C-PC treatment [53]. This finding aligns with our observation that *Spirulina* protein can induce apoptosis in colon cancer cell lines, potentially through similar mechanisms. The differences in apoptotic responses between the two cell lines in our study may warrant additional investigation into the molecular pathways involved and whether *Spirulina* protein’s effects are mediated through different signaling mechanisms in various cancer cell types.

In summary, the results indicate that *Spirulina* protein exhibits potent anticancer effects against Caco-2 and HT-29 cells, likely through apoptosis induction, oxidative stress generation, and cell cycle arrest, while maintaining a lower cytotoxic effect on normal Vero cells. These findings highlight the potential of *Spirulina* protein as a natural anticancer agent and warrant further investigation into its molecular mechanisms, bioactive components, and possible applications in cancer therapy.

### 3.5. Functional Properties

The functional properties of proteins play a crucial role in determining their applicability in food and biomedical industries. The functional characteristics of proteins are largely influenced by their interactions with key food system components, particularly oil and water. Acting as surface-active agents, proteins contribute significantly to interfacial properties, with their functionality being determined by how effectively they adsorb at water–oil or oil–water interfaces. The extracted protein from *Spirulina* exhibited notable solubility, foaming, emulsifying, and digestibility characteristics (Table 5).

#### 3.5.1. Solubility

Protein solubility is a key determinant of its functionality in various applications. The solubility behavior of a protein is primarily determined by its interaction with water. The extracted protein from *Spirulina* exhibited a solubility of 3.70%, which is relatively low compared to other plant-based proteins. The low solubility of the extracted *Spirulina* protein could be attributed to precipitation at pH 4.0 during extraction. This pH is close to the isoelectric point (pI) of many algal proteins [54], where the net charge on protein molecules is minimized, reducing electrostatic repulsion and leading to aggregation and precipitation. At the isoelectric point, protein–protein interactions dominate over protein–water interactions, resulting in decreased solubility. The precipitation at pH 4.0 suggests that the extraction conditions favored protein aggregation, which may have limited the availability of hydrophilic functional groups for interaction with water. Our results align with previous findings that microalgal proteins exhibit reduced solubility at pH values below 5 [55]. However, modifications such as pH adjustments or enzymatic hydrolysis could enhance solubility, improving its applicability in food formulations. In its current state, the extracted *Spirulina* protein’s low solubility makes it unsuitable for direct use in beverages. However, with appropriate modifications, its solubility can be improved to meet the requirements for beverage applications.

#### 3.5.2. Foaming Properties

Foaming capacity (FC) and foaming stability (FS) are critical for applications in aerated food products. Foam is a biphasic system composed of air cells enclosed within a thin, continuous liquid layer known as the lamellar phase [56]. The foam produced from *Spirulina* protein isolates exhibited greater density and stability, likely due to enhanced interactions at the air–water interface [57]. The extracted *Spirulina* protein demonstrated an FC of 100%, indicating a strong ability to entrap air and form a stable foam matrix. Additionally, the foam stability was 98.01%, suggesting that the formed foams remained intact over time with minimal collapse. The elevated foaming capacity and stability suggest that the foam structure retains a significant amount of water, leading to well-hydrated foams. The superior foaming characteristics of *Spirulina* protein may be attributed to its unique amino acid composition, particularly its hydrophobic residues, which enhance air entrapment and film stability. Our results can be attributed to its high hydrophobic amino acid content (50.11% of total amino acids). These attributes are largely influenced by its unique amino acid composition. This aligns with previous findings that *Spirulina* contains high levels of hydrophobic amino acids which enhance air entrapment and stabilize foam structures [58]. These results indicate that *Spirulina* protein could be a potential ingredient in food formulations requiring high foaming ability, such as whipped toppings and bakery products.

#### 3.5.3. Emulsifying Properties

The emulsifying capacity (EC) and emulsifying stability (ES) are critical functional properties of proteins in stabilizing oil–water interfaces, particularly in food and pharmaceutical formulations. The high EC (94.05%) of the extracted protein suggests that it possesses a strong interfacial activity, enabling the formation of fine and stable emulsions. This property is often attributed to the amphiphilic nature of proteins, where hydrophilic and hydrophobic regions facilitate adsorption at the oil–water interface, reducing interfacial tension and promoting emulsion formation [59]. Additionally, the ES (92.86%) indicates the protein’s ability to maintain emulsion stability over time, preventing phase separation or coalescence.

The high emulsifying properties observed in this study may be influenced by several structural and compositional factors, including the protein’s molecular weight distribution and surface hydrophobicity. Proteins with flexible structures, moderate hydrophobicity, and sufficient solubility are generally more effective emulsifiers. Low-molecular-weight proteins (10–25 kDa) in our study (Figure 2) are generally more surface-active due to their higher flexibility and ability to diffuse rapidly to the oil–water interface [60]. The presence of specific amino acids such as glutamic acid, aspartic acid, and hydrophobic residues contribute to interfacial stabilization by enhancing protein adsorption and structural rearrangement at the interface. Mechanistically, the superior EC and ES of the extracted protein may be linked to its structural integrity and the ability to form a viscoelastic interfacial film, which resists external stresses such as shear forces and temperature fluctuations [61]. Compared to conventional emulsifiers like casein and whey protein, which rely on their globular structure and surface-active peptides for stabilization [62], the extracted protein in this study might provide an alternative plant-based or microalgae-based emulsifier with comparable or even superior performance. Further studies on interfacial rheology, zeta potential, and protein-unfolding behavior would provide deeper insights into the mechanism governing its emulsifying properties. These properties suggest that *Spirulina* protein can be effectively utilized in salad dressings, dairy alternatives, and other emulsion-based products.

#### 3.5.4. Digestibility

The digestibility of *Spirulina* protein was recorded at 85.77%, highlighting its efficient breakdown and absorption in the gastrointestinal tract. This high digestibility can be attributed to the structural properties of *Spirulina* proteins, including their amino acid composition, molecular weight distribution, and susceptibility to enzymatic hydrolysis. *Spirulina* proteins are predominantly globular, with a balanced ratio of hydrophilic and hydrophobic amino acids, allowing for efficient enzymatic cleavage [63]. The presence of low-molecular-weight proteins enhances digestibility since smaller proteins and peptides are more easily broken down by digestive enzymes. Our study result of 85.77% digestibility for *Spirulina* protein falls between the high and low values reported for *A. platensis*. While some studies found a digestibility of 94% [64], others indicated that the digestibility of microalgae proteins is lower when compared to conventional protein sources such as egg, soy, and pea protein. Specifically, digestibility values for *A. platensis* suggest a much lower digestibility (78%) [65]. Our study suggests that the digestibility of *Spirulina* protein is somewhat higher than the lower value found for *A. platensis* but lower than the highest value, possibly indicating that *Spirulina* has a moderate digestibility in comparison to other microalgae species. The in vitro protein digestibility (IVPD) of brown and red seaweeds ranging between 78% and 88% [66] is slightly higher than the digestibility of *Spirulina* protein reported in our study. Both *Spirulina* and these seaweeds fall within the same range of high digestibility, indicating that they are both efficiently broken down and absorbed in the gastrointestinal tract. The high digestibility suggests that the extracted protein could serve as an excellent alternative protein source in functional foods and nutraceutical applications. *Spirulina*, as a blue-green algae, tends to have a more favorable protein structure for digestion, which might explain its high digestibility. Its cell wall is softer, making it more accessible to digestive enzymes compared to some other seaweeds, which may have more rigid cell walls or higher levels of indigestible polysaccharides.

The functional properties of the extracted *Spirulina* protein indicate its potential as a sustainable and versatile protein source. Its excellent foaming and emulsifying properties suggest applications in food formulations, while its high digestibility supports its use in nutritional products. Future studies may focus on improving solubility through structural modifications and exploring its bioactivities to expand its potential applications in biomedical and pharmaceutical fields. Furthermore, future studies focusing on protein purification could provide a more precise characterization of its functional properties and enhance its applicability in specific industrial applications.

## 4. Conclusions

This study demonstrates the effectiveness of ultrasound-assisted deep eutectic solvent (DES) extraction in isolating bioactive proteins from *Spirulina* biomass. The optimized method successfully enhanced protein yield and preserved essential bioactive properties. The extracted proteins exhibited strong antioxidant activity and selective cytotoxicity against colorectal cancer cell lines, highlighting their potential for pharmaceutical applications. Functional characterization showed promising solubility, foaming, and emulsifying properties, making the extract suitable for food and nutraceutical industries. The method’s efficiency and sustainability position it as a viable alternative to conventional extraction techniques. Future research should focus on scaling up the process and evaluating its clinical efficacy to fully harness the therapeutic potential of *Spirulina*-derived proteins.

## Figures and Tables

**Table 1:** Experimental design and results of protein yield from *Spirulina* biomass after ultrasound-assisted deep eutectic solvent (DES) extraction.

Run	Biomass Concentration (%)	Amplitude (%)	Time (minutes)	Protein Yield (%)	
				Experimental Value	Predicted Value
1	3	30	30	68.53 ± 1.19	68.31
2	3	30	55.23	75.73 ± 1.31	74.79
3	3	30	30	67.45 ± 1.11	68.31
4	5	40	45	88.09 ± 0.09	88.29
5	3	30	30	68.98 ± 1.21	68.31
6	1	20	15	54.86 ± 0.12	54.56
7	3	30	4.77	60.54 ± 1.40	61.63
8	3	13.18	30	70.23 ± 0.51	70.38
9	6	30	30	70.72 ± 1.80	70.87
10	1	40	15	68.60 ± 0.98	68.01
11	5	40	15	83.29 ± 0.90	82.99
12	5	20	15	66.18 ± 0.93	65.46
13	5	20	45	69.43 ± 1.51	69.92
14	1	40	45	78.58 ± 0.86	79.19
15	1	20	45	64.70 ± 1.14	64.90

**Table 2:** Analysis of variance (ANOVA) for the response surface quadratic model of protein yield from *Spirulina* biomass after ultrasound-assisted deep eutectic solvent (DES) extraction.

Source	Sum of Squares	df	Mean Square	F-Value	*p*-Value	
Model	976.84	9	106.28	106.28	<0.0001	Significant
A	218.35	1	213.81	213.81	<0.0001	Significant
B	552.51	1	552.51	541.02	<0.0001	Significant
C	208.93	1	208.93	204.58	<0.0001	Significant
AB	8.30	1	8.30	8.13	0.0358	Significant
AC	17.32	1	17.32	16.96	0.0092	Significant
BC	0.36	1	0.36	0.35	0.5801	Not significant
A^2^	28.22	1	28.22	27.63	0.0033	Significant
B^2^	196.28	1	196.28	192.20	<0.0001	Significant
C^2^	0.014	1	0.014		0.9110	Not significant
Residual	5.11	5	1.02			
Lack of Fit	3.87	3	1.29	2.09	0.3403	Not significant
Pure Error	1.24	2	0.62			
Cor Total	981.95	14				
R^2^	0.9948					
Adequate Precision	40.882					

Note: A is biomass concentration, B is amplitude, and C is sonication time.

**Table 3:** Protein content, total phenolics, and total sugar of extracted protein.

Parameters	Extracted Protein from *Spirulina*
Protein concentration (mg/g extract)	442.88 ± 13.70
Total phenolic GAE (mg gallic acid/g extract)	4.87 ± 2.34
Total sugar (mg/mL)	0.18 ± 0.03

**Table 4:** Amino acid composition of *Spirulina* protein.

Amino Acids (g/100 g)	Protein Extract	WHO/FAO/UNU	
		Children	Adult
Aspartic acid	3.18	NA	NA
Threonine ^a^	1.92	2.5	2.3
Serine	2.91	NA	NA
Glutamic acid	10.38	NA	NA
Proline	0.33	NA	NA
Glycine	1.05	NA	NA
Alanine	4.16	NA	NA
Cysteine	0.04	NA	NA
Methionine ^a^	0.36	2.4	2.2
Valine ^a^	2.46	4.0	3.9
Isoleucine ^a^	2.52	3.1	3.0
Leucine ^a^	7.32	6.1	5.9
Tyrosine	2.08	NA	NA
Phenylalanine ^a^	2.88	4.1	3.8
Histidine ^a^	0.63	1.6	1.5
Lysine ^a^	0.33	4.8	4.5
Arginine	3.70	NA	NA
Acidic ^b^	29.27		
Basic ^b^	10.07		
Hydrophilic ^b^	38.62		
Hydrophobic ^b^	50.11		
EAA ^b^	39.91		

^a^ Essential amino acids (EAAs); ^b^ Relative to total amino acids; NA is not available; Acidic: aspartic acid, glutamic acid; Basic: arginine, histidine, lysine; Hydrophilic: aspartic acid, threonine, serine, glutamic acid, lysine, histidine, arginine; Hydrophobic: valine, methionine, isoleucine, leucine, phenylalanine, proline, glycine, alanine, cysteine, tyrosine.

**Table 5:** Functional properties of extracted *Spirulina* protein.

Functional Properties	Extracted Protein from *Spirulina*
Solubility	3.70 ± 0.10%
Foaming capacity (FC)	100 ± 0%
Foaming stability (FS)	98.01 ± 0.14%
Emulsifying capacity (EC)	94.05 ± 1.69%
Emulsifying stability (ES)	92.86 ± 0.00%
Digestibility (%)	85.77 ± 0.30%

## Data Availability

The original contributions presented in the study are included in the article; further inquiries can be directed to the corresponding author.
